# Microvascular Adaptations to Muscle Stretch: Findings From Animals and the Elderly

**DOI:** 10.3389/fphys.2022.939459

**Published:** 2022-07-04

**Authors:** Kazuki Hotta, Judy Muller-Delp

**Affiliations:** ^1^ Department of Rehabilitation Sciences, Graduate School of Medical Sciences, Kitasato University, Sagamihara, Japan; ^2^ Department of Rehabilitation, Kitasato University School of Allied Health Sciences, Sagamihara, Japan; ^3^ Department of Biomedical Sciences, Florida State University College of Medicine, Tallahassee, FL, United States

**Keywords:** muscle stretch, microvascular endothelial function, skeletal muscle, nitric oxide, blood flow, elderly

## Abstract

Microcirculation in skeletal muscle is disturbed with advancing aging, causing limited capillary blood flow and exercise incapacity. Muscle stretch has been widely performed in physical therapy, sports medicine, and health promotion. However, the effect of stretch on microvascular reactivity and muscle blood flow remains unknown. This review focuses on stretch-induced microvascular adaptations based on evidence from cultured cells, small animals, and human studies. Vascular endothelium senses and responds to mechanical stimuli including stretch. This endothelial mechanotransduction potentially plays a vital role in the stretch-induced microvascular adaptation alongside hypoxia. Aging impairs microvascular endothelial function, but muscle stretch has the potential to restore it. Muscle stretch may be an alternative to improve vascular function and enhance exercising blood flow, especially for those who have difficulties in participating in exercise due to medical, functional, or psychological reasons.

## Introduction

As a “super-aged” society, Japan has the fastest aging population in the world, and COVID-19 has had a striking impact on the physical activity of the elderly. It has been reported that the total physical activity time of older adults who were living alone and socially inactive decreased by approximately 40–60% after the pandemic began (Yamada et al., 2021). Even before the pandemic, primary prevention of cardiovascular disease was an important medical policy. Non-pharmacological vasoprotective strategies, especially those implemented at home, are a high priority. Aerobic exercise training, such as treadmill exercise, may reverse age-related loss of exercise capacity (Fitzgerald et al., 1997); however, aerobic exercise often cannot be performed by the elderly due to its strenuous nature. Residents living in cities under lockdown or subjects with extreme exercise intolerance often have difficulty performing aerobic exercise. One of the methods of stimulating skeletal muscles ,that is, simple and does not require expensive equipment is muscle stretch. Muscle stretch has been widely performed in subjects who undergo physical therapy (Kay and Blazevich, 2012; Afonso et al., 2021; Gomez-Cuaresma et al., 2021). In the past, muscle stretch has been positioned as a control treatment against exercise training groups in many studies (Chubak et al., 2006; Iyalomhe et al., 2015; Best et al., 2018; Cho et al., 2018; Lopes et al., 2019; Thomas et al., 2020; Andrade-Lima et al., 2021; de Carvalho et al., 2021; Ngwa et al., 2021; Qi et al., 2021). It is worth noting; however, that stretch has a significant effect on blood vessels, and rather surprisingly, induces more favorable effects on large arteries than regular resistance training (Cortez-Cooper et al., 2008). Our clinical and animal studies have indicated stretch-induced microvascular adaptations (Hotta et al., 2013; Hotta et al., 2018; Hotta et al., 2019). Large arteries are involved in blood flow regulation, but even small vessels with less than 100 µM of diameter play a central role in the regulation of blood flow distribution in skeletal muscle (Fronek and Zweifach, 1975). Therefore, this review will focus on stretch-induced microvascular adaptation based on evidence from cultured cells, animals, and human.

## Mechanisms of Sensing Stretch Stimuli in Blood Vessels

**FIGURE 1:**
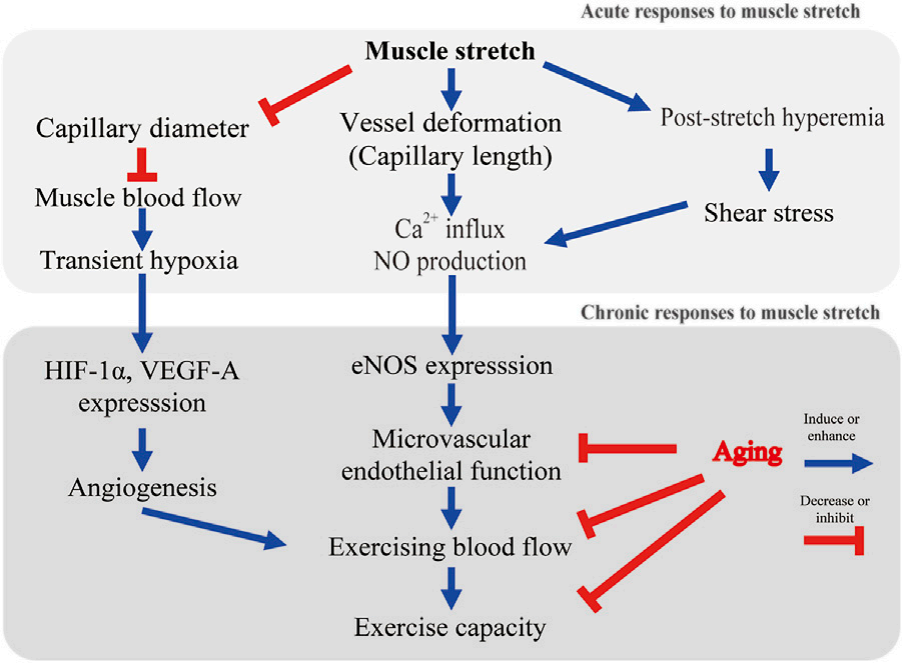
Possible mechanisms of stretch-induced microvascular adaptation. Ca^2+^, calcium ion; NO, nitric oxide; HIF-1α, hypoxic inducible factor-1α; eNOS, endothelial nitric oxide synthase.

Before considering vascular adaptations to muscle stretch, the mechanisms of sensing mechanical stimuli and intracellular signaling triggered by mechanical stimuli should be mentioned. Vascular endothelial cells constitute the inner layer of blood vessels and are constantly exposed to blood flow. Skeletal muscle senses and responds to mechanical stimuli. Armstrong et al. reported that stretch of rat soleus muscle results in calcium ion (Ca^2+^) influx into myocytes from the extracellular space (Armstrong et al., 1993). Blood vessels also sense mechanical stimuli and transmit this signal into the cell. It has recently become apparent that cultured endothelial cell functions are controlled by hemodynamic forces such as shear stress and stretch (Ito et al., 2010; Ando and Yamamoto, 2011). Intracellular Ca^2+^ increases rapidly when a vascular endothelial cell is stretched by a flexible membrane (Naruse and Sokabe, 1993). The intensity of stretch on endothelial cells has been found to affect the degree of intracellular Ca^2+^ response (Naruse and Sokabe, 1993). Stretch-induced Ca^2+^ influx triggers the production and release of nitric oxide (NO, vasorelaxant factor) from endothelial cells (Naruse and Sokabe, 1993; Takeda et al., 2006; Ito et al., 2010). Cyclic stretch alone increased endothelin-1 mRNA levels, but had no effect on endothelial NO synthase (eNOS) mRNA levels in cultured endothelial cells (Toda et al., 2008). Another study reported that cyclic stretch increases the expression of eNOS transcripts and protein levels (Awolesi et al., 1995). Interestingly, focusing on the magnitude of the stretch of vascular endothelial cells (i.e., the degree of cellular deformation), eNOS expression was increased in the 10% strain condition compared to the 6% condition (Awolesi et al., 1995). These studies indicate that endothelial cells play a mechanosensitive role and potentially regulate eNOS and blood flow *in vivo* (Figure 1).

## Microvascular Morphological and Hemodynamic Responses to Muscle Stretch

Muscle stretch induces hemodynamic changes in capillary blood flow. Capillaries are aligned parallel to the long axis of the muscle cell. Studies using *in vivo* imaging have shown that capillary red blood cell velocity and flux decrease as muscle sarcomere length increases (Kindig and Poole, 2001). This response is a striking contrast to the rapid capillary blood flow increase during muscle contractions (Kindig et al., 2002). Limited capillary blood flow in stretched muscle probably results from narrowing of the capillary diameter (Mathieu-Costello, 1987; Ellis et al., 1990; Kindig and Poole, 2001). In addition to a decrease in vessel diameter, capillary length is extended, and tortuosity is reduced by muscle stretch (Mathieu-Costello, 1987; Ellis et al., 1990; Poole et al., 1997). Ellis et al. conducted *in vivo* imaging to evaluate the relationship between the change in sarcomere and capillary length (interbranch distances) (Ellis et al., 1990). Capillaries running parallel to the long axis of the muscle fiber are tortuous. Muscle extension decreases the tortuosity, leaving vessel length unaltered (Ellis et al., 1990). Once capillaries are pulled into a straight configuration, further extension of the muscle causes the vessels to stretch. Ellis et al. showed some capillaries stretched to the same degree as the muscle, but others stretched more or less (Ellis et al., 1990). It remains unknown whether capillaries running in the short axis of the muscle or arterioles are morphologically altered by stretching.

In our animal model, dorsiflexion splints were applied to a rat ankle joint. Blood flow reduction was observed during stretching in aged rats (Hotta et al., 2018). This blood flow reduction was found in plantar flexor muscles (soleus, plantaris, flexor digitorum longus, and flexor hallucis longus), but not in dorsiflexor muscles (tibialis anterior or extensor digitorum longus) (Hotta et al., 2018). Skeletal muscle blood flow during stretching was reduced to about 60% of the resting level (Hotta et al., 2018). This result is consistent with the previous human study that showed femoral blood flow and conductance were significantly decreased during 45 s of passive muscle stretch (Venturelli et al., 2019). Thus, blood flow is acutely decreased with muscle elongation. The external compression resulting in intramuscular pressure increase may be a possible factor that contributes to blood flow reduction during stretching (Sejersted et al., 1984; Ameredes and Provenzano, 1997). Although red blood cells in the capillaries are the main oxygen carriers, it is not known whether decreased blood flow induces muscle hypoxia during stretching. Using near-infrared spectroscopy, Kruse et al. found an increase in deoxygenated hemoglobin in stretched human muscle (Kruse et al., 2016; Kruse and Scheuermann, 2016). In our animal model, hypoxic inducible factor-1α (HIF-1α) was expressed in stretched muscle (Hotta et al., 2018). Therefore, we cannot rule out the stretch-induced reduction in muscle oxygenation. Fuse et al. reported an increase in muscle tissue oxygen saturation without any changes in cardiac output as a result of passive cycling exercise in healthy adult males (Fuse et al., 2016). Passive cycling exercise (50 rpm) applies a cyclic mechanical stimulus to the skeletal muscles of the lower extremities. Interestingly, it was reported that passive cycling did not alter the blood flow in the common femoral arteries (Fuse et al., 2016). These studies indicate that static stretch may decrease muscle blood flow (Figure 1), while cyclic stretch possibly increases muscle capillary blood flow but not blood flow in large arteries. In future studies, direct measurement of the partial pressure of oxygen during stretching may assist in understanding this effect.

Kruse et al. found enhanced blood flow immediately after stretch (post-stretch hyperemia) in healthy male subjects (Kruse et al., 2016) (Figure 1). Post-stretch hyperemia has been also shown in healthy male subjects in a study by Venturelli and colleagues (Venturelli et al., 2019). In elderly patients with acute myocardial infarction, tissue oxygen pressure at the thigh was found to increase after a single session of muscle stretch (Hotta et al., 2013). Contrary to the previous human studies, our animal study did not show post-stretch hyperemia (Hotta et al., 2018). We performed 30 min of dorsiflexion splinting on one leg of old rats and measured muscle blood flow for comparison between stretched and contralateral limbs (Hotta et al., 2018). Kruse et al. performed 4 min, and Venturelli et al. performed 45 s of static stretch, and both studies demonstated post-stretch hyperemia (Kruse et al., 2016; Kruse and Scheuermann, 2016; Venturelli et al., 2019). Bisconti et al. showed a significant increase in arterial shear stress; however, it gradually decreased during repeated cycles of 45 s of stretch/15 s of relaxation in healthy young adults (Bisconti et al., 2020). Stretch duration possibly affected post-stretch reactive hyperemia. Further studies are needed to determine the stretch duration that produces the greatest post-stretch reactive hyperemia.

## Effect of Aging and Daily Muscle Stretch on Hyperemic Response During Exercise

Aging reduces blood flow to the high-oxidative red skeletal muscle; however, exercise training increases blood flow and vascular conductance during exercise (Behnke et al., 2012). Therefore, exercise training may contribute to correcting the balance between oxygen demand and supply in skeletal muscle (McCullough et al., 2011; Hirai et al., 2012). While there are undoubtedly positive effects of exercise training, there is a need to provide non-exercise options for the elderly who have difficulty participating in the exercise.

In our model of old rats, although there was no effect on resting blood flow, a significant improvement in exercising blood flow was found after 4 weeks of daily dorsiflexion splinting (Hotta et al., 2018) (Figure 1). These results were found only in muscles that had been stretched (soleus, plantaris, flexor digitorum longus, and flexor hallucis longus), but not in the contralateral limb or plantar flexor muscles (tibialis anterior, extensor digitorum longus) (Hotta et al., 2018). This suggests that muscle stretch enhanced microvascular reactivity and regulated local muscle blood flow. A most recent randomized controlled trial revealed that 4 weeks of daily passive leg movement increased the blood flow capacity of the common femoral artery in chronically bedridden people aged 87 years (Pedrinolla et al., 2022). Although the microcirculatory dynamics within the skeletal muscle are not known, it is noteworthy that daily stretching of the thigh muscles contributed to improved blood flow in feed arteries even in elderly subjects who were bedridden for 4 years.

## Microvascular Adaptations to Daily Muscle Stretching in the Elderly

Aging impairs endothelium-dependent vasodilation in rat skeletal muscle arterioles (Figure 1) (Muller-Delp et al., 2002). Exercise training reverses microvascular endothelial dysfunction in skeletal muscle of aged rats (Spier et al., 2004). To test our hypothesis that daily stretching improves skeletal muscle arteriolar function, a four-week course of daily splinting was performed in aged rats (Hotta et al., 2018). A dorsiflexion splint was applied to produce stretching of dorsiflexor muscles on only one hindlimb of the aged rats, and the contralateral side was used as a non-stretched limb. At the end of the four-week course of stretching, arterioles from the soleus muscle were isolated, cannulated, and pressurized to evaluate acetylcholine-induced vasodilatory responses. Compared to arterioles from the control limb, the acetylcholine (ACh)-induced vasodilatory response was enhanced in arterioles from the stretched limb; however, inhibition of eNOS with L-NAME dramatically reduced ACh-induced vasodilation and eliminated differences in responsiveness between arterioles from the stretched and control limbs. These results supported our hypothesis that muscle stretch enhances NO-dependent vasodilatory responses in skeletal muscle arterioles of aged rats (Figure 1).

Awolesi et al. reported increased eNOS in cultured endothelial cells after 24 h of cyclic stretch stimuli (Awolesi et al., 1995). Thacher et al. developed interesting *ex vivo* experiments to evaluate the relationship between stretch and eNOS expression (Thacher T. et al., 2010; Thacher T. N. et al., 2010). They reported blunting of endothelium-dependent vasodilation of isolated carotid arteries maintained without stretch for 24 h compared to 24 h of longitudinal cyclic stretch (Thacher T. et al., 2010; Thacher T. N. et al., 2010). They also reported that the phosphorylation of serine-1177 on eNOS was decreased, and reactive oxygen species were increased in vessels not exposed to cyclic stretch. Consistent with these results, our study indicated that passive muscle stretch increases eNOS expression in intramuscular arterioles of aged rats (Figure 1). Considering the blood flow reduction during muscle stretching, muscle stretch may have different mechanisms to exercise-induced microvascular adaptation.

Our study was the first to report stretch-induced skeletal muscle angiogenesis of aged rats (Figure 1). Capillarity was higher in the soleus muscle of the stretched limb compared to the contralateral limb (Hotta et al., 2018). Capillary volume in the soleus muscle was also significantly higher compared to the contralateral limb (Hotta et al., 2018). Vascular endothelial cells express HIF-1α when exposed to hypoxia (Levy et al., 1995; Westvik et al., 2009). It has been reported that stimulation of skeletal muscle also expresses HIF-1α and downstream vascular endothelial growth factor (Holly et al., 1980; Milkiewicz et al., 2001; Rivilis et al., 2002). HIF-1α and vascular endothelial growth factor are angiogenic factors and increase after a single bout of blood flow-restricted exercise (Gustafsson et al., 1999) or with sustained stretch (Milkiewicz et al., 2007). Local areas of ischemia and hypoxia may have contributed to increased HIF-1α. A more probable mechanism driving the upregulation of HIF-1α is the prolonged deformation of blood vessels (Figure 1). Chang et al. reported that cyclical mechanical stretch increased HIF-1α gene expression in cultured vascular smooth muscle cells (Chang et al., 2003). In isolated vessels, prolonged stretch is associated with HIF-1α expression and progressive vessel dilation (Lim et al., 2011). Stretch-induced angiogenesis may partially explain the enhanced exercise hyperemia after daily stretching (Figure 1).

## Effect of Muscle Stretching on Exercise Capacity in the Elderly

A key mechanism for the age-related reduction in exercise capacity (Figure 1) is an oxygen delivery-to-demand mismatch resulting from alterations in skeletal muscle blood flow (Behnke et al., 2005). This review provides a novel method of muscle stretch to improve exercise hyperemia in aged skeletal muscle. Stretching is a type of exercise therapy that has been practiced for many years, but the corresponding vascular adaptation has been newly elucidated in the past several years (Hotta et al., 2013; Hotta et al., 2018; Kato et al., 2020). In young adults, 8-weeks of passive muscle stretch alone did not improve peak oxygen consumption, but 8-weeks of active knee extension improved peak oxygen consumption by 3.0 ml/kg/min (Ce et al., 2022). In the elderly, prevention of frailty is expected to prolong life expectancy and reduce medical and nursing care costs. There is no evidence that muscle stretch improves exercise capacity in older adults with frailty. Rehabilitation comprising of muscle stretch, muscle strengthening, balance training, and endurance exercise including walking has been reported to increase the activity of daily living scores in elderly (>80 years) patients with heart failure (Obata et al., 2021). However, the effect of muscle stretch alone remains unknown. Our study evaluated the clinical effects of passive calf muscle stretching on popliteal arterial function and walking capacity in elderly patients with symptomatic peripheral artery disease (PAD) (Hotta et al., 2019). All patients enrolled in this study had significant difficulty in walking (e.g., required the use of grocery store carts due to limitations in walking). Participation in exercise may have been beneficial, as guidelines recommended a supervised or home-based treadmill exercise program (Treat-Jacobson et al., 2019); however, treadmill exercise is challenging due to severe exercise limitations. In our study, daily calf muscle stretching, accomplished using a simple dorsiflexion brace, was conducted without participant drop-out (Hotta et al., 2019). Passive stretching was associated with significant improvements in endothelium-dependent vasodilation (flow-mediated dilation) and walking distance on the six-minute walking test (Hotta et al., 2019). Although the mechanisms underlying the walking incapacity of patients with PAD remain unknown, alterations in skeletal muscle blood flow and insufficient oxygen supply to active muscle appear to be critical determinants (Robbins et al., 2011). In patients with PAD, Robbins et al. indicated that capillary density correlated with walking distance (Robbins et al., 2011). Our data showed a positive correlation between vascular endothelial function and walking capacity in elderly patients with PAD. Therefore, muscle stretch may be helpful as a vascular therapy for patients with cardiovascular disease. However, muscle stretch rather than aerobic exercise in rehabilitation has not been studied. There are many uncertainties, and more evidence regarding its application in elderly patients is needed. Muscle stretching has been shown to increase joint range of motion but not improve muscle thickness, strength or exercise tolerance (Longo et al., 2021; Ce et al., 2022). Further validation by RCTs is needed to verify the effects of stretching on exercise tolerance and motor function.

## Discussion

In this review, we have discussed the effects of muscle stretch on the microvasculature, primarily in the elderly. A conceptual diagram of the effects of muscle stretch on skeletal muscle microcirculation is shown in Figure 1. Endothelial cells sense mechanical stimuli in milliseconds, triggering an immediate intracellular response. This mechanoreception and intracellular signaling were considered to be the earliest responses induced by stretch. During the stretch, which lasts from a few seconds to several minutes, skeletal muscle blood flow is markedly reduced. Reactive hyperemia may occur during relaxation (Kruse et al., 2016; Venturelli et al., 2019), but this remains controversial. Future research should focus on the duration of muscle stretch, since the duration may affect the microcirculatory dynamics during stretching (Bisconti et al., 2020). A relatively large number of studies have demonstrated the effect of aerobic exercise training on vascular endothelial function (Ashor et al., 2015). Still, fewer studies have examined the effects of daily muscle stretch (Kato et al., 2020). A recent meta-analysis reported by our group suggested that muscle stretch reduces arterial stiffness and improves vascular endothelial function in middle-aged and older adults (Kato et al., 2020). Kato et al. applied muscle stretch training for patients with chronic heart failure (CHF) and an implantable cardioverter defibrillator (ICD) or cardiac resynchronization therapy-defibrillator (CRT-D) (Kato et al., 2017). Aerobic exercise training improves vascular endothelial function in patients with CHF; however, patients with an ICD or CRT-D often avoid aerobic exercise training for fear of ICD shock. Kato et al. showed that 4 weeks of muscle stretch improved vascular endothelial dysfunction through attenuation of oxidative stress in sedentary patients with CHF and an ICD or CRT-D (Kato et al., 2017). Pedrinolla et al. successfully applied passive muscle stretch to individuals of advanced age who are chronically bedridden and found improvement of arterial flow capacity (Pedrinolla et al., 2022). Considering these recent randomized and non-randomized controlled trials (Kato et al., 2017; Kato et al., 2020; Pedrinolla et al., 2022), stretching may be a good alternative for those who are bedridden due to chronic illness or who cannot participate in aerobic exercise due to medical, functional, or psychological reasons. Passive stretching with braces (Hotta et al., 2019) or self-administered active stretching (Kato et al., 2017) at home may be an effective alternative. To achieve more significant functional improvement, aerobic exercise or resistance exercise would need to resume in addition to muscle stretch. The benefits of muscle stretch are limited to the stretched muscles of aged rats (Hotta et al., 2018). In contrast, aerobic exercise has a positive effect on vascular function in both the active and inactive limbs (Birk et al., 2012). Therefore, it is assumed that a realistic adaptation of muscle stretch would be a bridge from exercise withdrawal to a return to aerobic exercise. Once muscle stretch has improved walking capacity, the frequency of muscle stretch training should be gradually reduced alongside an increase in aerobic exercise such as walking.

The effect of muscle stretch on inactive limbs is still controversial. A recent RCT conducted by Bisconti et al. (Bisconti et al., 2020) revealed two new findings of muscle stretch. First, stretching of the unilateral femoral and calf muscles improved popliteal arterial endothelial function in adults with an average age of 23 years (did not include the elderly), but also improved endothelial function of the upper limb (brachial) artery. They also showed a significant improvement in popliteal arterial endothelial function of the contralateral limb. Their findings were accompanied by a reduction of augmentation index and pulse wave velocity, which indicated an improvement in systemic vascular endothelial function and stiffness (Bisconti et al., 2020). These results suggest that muscle stretch induces a positive effect (similar to aerobic exercise) in the inactive limbs of healthy young adults. The reasons for the different results from Bisconti et al. and our group could be the differences in species (human or rat), age (young or old), and muscle groups to be stretched (femoral and/or calf muscles). Second, they evaluated the de-training effect of muscle stretch (Bisconti et al., 2020). Six-week of de-training returned endothelial function in the inactive limb to the same level as before training, but preserved arterial function in the lower limb that had received the stretching stimulus (Bisconti et al., 2020). A recent study comparing the effects of muscle stretch and resistance training found that muscle stretch had an equal effect on vascular endothelial function but had smaller or no effects on thigh blood flow, knee extensor strength, and exercise tolerance compared to resistance training (Ce et al., 2022). Considering all of these studies together, it is assumed that the effect on arteries in the limb not receiving the stretch stimulus is limited or diminished after the cessation of muscle stretch. Therefore, as mentioned above, gradually increasing the amount of physical activity in daily life may be beneficial in maintaining systemic vascular health. In fact, it may not be easy for the frail elderly to stretch their own femoral and calf muscles for 30–40 min every day, but it would be more realistic for them to walk indoors or outdoors in their daily lives. And again, if the elderly are capable of active muscle contractions, then they should perform spontaneous exercises rather than passive muscle stretch.

The present review focused on the microvascular adaptations to acute and chronic muscle stretch in the elderly. The current findings suggest that muscle stretch improves endothelium-dependent vasodilation in the skeletal muscle microvasculature of aged rats, which alters blood flow responses to exercise and solves the oxygen supply and demand problem which occurs with advancing age. The microvascular adaptations to stretch appear to be seen mainly in stretched muscle, but there may be a crossover effect on the vascular endothelial function to the opposite side or to the inactive limbs. In addition to functional changes, structural changes such as stretch-induced angiogenesis and muscle hypertrophy may occur in old rats (Hotta et al., 2018); however, in young adults, no muscle hypertrophy was observed with muscle stretch (Ce et al., 2022). From basic mechanisms to clinical research, the efficacy of stretching in elderly patients with acute myocardial infarction, CHF, and PAD has been investigated, and positive effects on vascular endothelial function have been reported. Future work will be needed to elucidate the contribution of mechanical stimuli and ischemia to stretch-induced microvascular adaptations and the clinical impact of muscle stretch in cardiovascular medicine. It is hoped that more studies will examine the effects of muscle stretch on microvasculature and exercise capacity in the elderly.
